# Anticoagulation and thromboembolic risk in critically ill patients with trigger-induced atrial fibrillation—A systematic review and meta-analysis

**DOI:** 10.1007/s12471-025-01978-9

**Published:** 2025-08-28

**Authors:** Jasper Koolwijk, Mileen van de Kar, Brittney A van der Woude, Marcel van ’t Veer, Harm Jan de Grooth, Harry J. G. M. Crijns, Lukas R. C. Dekker, R. Arthur Bouwman, Olaf L. Cremer, Ashley J. R. de Bie, Luuk C. Otterspoor

**Affiliations:** 1 Department of Anesthesiology, Intensive Care and Pain Medicine, Catharina Hospital Eindhoven, Eindhoven, The Netherlands; 2 Department of Cardiology and Cardiothoracic Surgery, Catharina Hospital Eindhoven, Eindhoven, The Netherlands; 3 Intensive Care Center, University Medical Center Utrecht, Utrecht, The Netherlands; 4 Department of Cardiology, Maastricht University Medical Center, Cardiovascular Research Institute Maastricht, Maastricht, The Netherlands; 5 Department of Electrical Engineering, Eindhoven University of Technology, Eindhoven, The Netherlands; 6 Department of Intensive Care, Catharina Hospital Eindhoven, Eindhoven, The Netherlands

**Keywords:** Atrial fibrillation, Stroke, Thromboembolism, Bleeding, Anticoagulation, Sepsis, Critical illness, Intensive care

## Abstract

**Introduction:**

In critically ill patients with trigger-induced atrial fibrillation, there are no definitive recommendations on the use of anticoagulation. This study aimed to evaluate the association between anticoagulation therapy and outcomes (i.e. thromboembolism, bleeding and mortality) and examine prescription patterns in high-risk individuals based on CHA_2_DS_2_-VASc scores.

**Methods:**

A systematic search was conducted to identify studies reporting on anticoagulation prescription, thromboembolism, bleeding, and mortality. Anticoagulation rates and CHA_2_DS_2_-VASc scores were correlated, and a meta-analysis was conducted to compare short- and long-term outcomes.

**Results:**

Anticoagulation prescription rates ranged from 3 to 86%; in over 50% of patients, CHA_2_DS_2_-VASc scores were ≥ 2 (*n* = 28 studies). A meta-analysis of eight observational studies, in which 95% of patients had sepsis/infection as the precipitant, demonstrated no association between anticoagulation and reduced short-term thromboembolism (OR 0.89, 95% CI 0.61–1.28) or increased bleeding (OR 1.05, 95% CI 0.90–1.22). Short-term mortality was lower in the anticoagulation group (OR 0.54, 95% CI 0.39–0.75), but a higher long-term thromboembolic risk was observed (OR 1.45, 95% CI 1.04–2.03).

**Conclusion:**

The prescription of anticoagulation in critically ill patients with TIAF is highly variable. There is no clear evidence of benefit or harm, and neither routine use nor systematic omission is supported.

**Supplementary Information:**

The online version of this article (10.1007/s12471-025-01978-9) contains supplementary material, which is available to authorized users.

## Introduction

Trigger-induced atrial fibrillation (AF) is a common complication during acute hospitalisation. The prevalence of trigger-induced AF in critically ill patients ranges from 6 to 46%, depending on disease severity [1]. According to current European and American guidelines, the standard of care for ambulatory patients with AF includes prevention of thromboembolic complications by weighing the risks and benefits of anticoagulation therapy [2–4], structured in risk scores such as CHA_2_DS_2_-VASc score and HAS-BLED [5, 6]. However, in patients with trigger-induced AF, these guidelines provide little or no guidance, often omitting a clear statement on the initiation of antithrombotic therapy during or after hospitalisation. Importantly, there is a paucity of data on the validity of these risk scores in this setting [7–9]. The lack of consensus may lead to a wide variety of clinical practice and potentially cause patient harm as a result of both under- and over-treatment with anticoagulants [10, 11].

The occurrence of trigger-induced AF in critical illness, such as sepsis, is linked to an elevated risk of stroke and increased mortality [12, 13]. It remains unclear whether stroke risk stems directly from AF or from factors such as acute illness, patient characteristics, or comorbidities. It is uncertain whether anticoagulant therapy can mitigate this increased stroke risk in this specific population in the short- and long-term. Moreover, given the potentially transient nature of trigger-induced AF in around half of these patients, clinicians face an additional challenge when prescribing long-term or even lifelong anticoagulation.

This review and meta-analysis aim to evaluate anticoagulation prescription rates and the short- and long-term effects of anticoagulation therapy on relevant outcomes, including thromboembolism, bleeding, and mortality, in trigger-induced AF in the critically ill patients. We hypothesize that the prescription of anticoagulation is highly variable and does not significantly affect thromboembolism, bleeding, or mortality rates.

## Methods

A systematic review was conducted in adherence with the Preferred Reporting Items for Systematic Reviews and Meta-Analyses (PRISMA) guidelines [14]. The protocol was registered with PROSPERO, an international prospective register of systematic reviews (ID: 561772).

### Database search

We conducted a comprehensive, systematic search of the electronic databases MEDLINE, Embase, and the Cochrane Library for all available literature up to March 2025. The search terms used for MEDLINE were: sepsis, critical care, atrial fibrillation, anticoagulants, stroke, bleeding, and mortality. Reference lists were also reviewed to identify additional eligible publications. The complete search strategy has been provided as Electronic Supplementary Material (ESM) (Table S1).

### Eligibility criteria

We considered publications that focused on patients with trigger-induced AF (also known as secondary new-onset atrial fibrillation) during acute hospitalisation [4]. This was defined as “new AF in the immediate association with a precipitating and potentially reversible factor”. We focused on patients with sepsis or those admitted to critical care units, as this condition and setting, respectively, are most likely to reveal an underlying predisposition to AF. We excluded studies on patients with pre-existing AF, trigger-induced AF due to chronic or cardiac illness (e.g., myocardial infarction), as well as primary cardiac surgery admissions.

The systematic review focused exclusively on longitudinal studies, including observational studies and controlled clinical trials. Brief communications and abstracts were included if they provided data on anticoagulation prescription. For the meta-analysis, only original studies reporting clinical outcomes (defined as the incidence of thromboembolic events, bleeding events, and mortality) stratified according to anticoagulation strategy were considered. Anticoagulation therapy was defined as any form of anticoagulation treatment, other than antiplatelet therapy or thromboembolic prophylaxis. Case series, case reports, reviews, and commentaries were excluded. Articles lacking original data or results contributing to the assessment of anticoagulation prescription and outcomes were omitted from consideration.

### Selection of eligible articles

Rayyan QCRI was utilized in the selection process after importing studies and the removal of duplicates [15]. Two authors (JK and MK) screened all titles and abstracts, retrieving full texts if criteria were met. Disagreements between reviewers were resolved by the decision of a third reviewer (LO). To avoid duplicates, we also identified studies that potentially shared a common population or data set.

### Outcomes and data extraction

The following outcomes were assessed: short- and long-term thromboembolism (or specifically stroke, if only stroke data were reported), bleeding, and mortality. Short-term outcomes were defined as outcomes that occurred during hospitalisation, and long-term outcomes were defined as outcomes that occurred after hospital discharge. The following data on study details were extracted: (first author, year), design, population, trigger-induced AF proportion, anticoagulation eligibility and prescription, and CHA_2_DS_2_-VASc score.

### Statistical analysis

The incidence of trigger-induced AF, the use of anticoagulation therapy, and clinical outcomes were analysed using descriptive statistics. The proportion of anticoagulated patients was defined as the number of patients receiving anticoagulation to treat trigger-induced AF divided by the total number of eligible patients, or, if this information was not available, by the total number of patients in the study. The proportion of patients with a CHA_2_DS_2_-VASc ≥ 2, which is the current absolute threshold for initiating anticoagulation therapy in accordance with current guidelines, was either directly extracted from the study or calculated using of the available data.

To conduct a meta-analysis, we utilized R statistics software (The R Foundation for Statistical Computing, Vienna, Austria) in conjunction with the “metafor” package. Heterogeneity among the included studies was quantified using I^2^ and Cochran’s Q test/Chi-square test. We defined heterogeneity as low (0–30%), moderate (30–60%), and considerable (60% or higher).

To mitigate the influence of small study effects, we employed a fixed-effect model to interpret the results, despite the heterogeneity observed among the studies [16]. Additionally, results from random-effects models were presented. We reported odds ratios/log odds ratios with 95% confidence intervals, considering a *p*-value of less than 0.05 as statistically significant. Forest plots were generated to illustrate the relative rates of clinical outcomes, including thromboembolic events, bleeding events and mortality.

### Risk of bias assessment

The Newcastle-Ottowa Scale (NOS) was used to assess the risk of bias for the included studies (JK and MK) [17]. Depending on the assessment studies were categorized as having a low, high or unclear risk of bias. Classification disagreements were resolved through discussion or a third reviewer (LO).

## Results

The search identified 6,448 publications from MEDLINE (*n* = 238), Embase (*n* = 6,086), and the Cochrane Library (*n* = 124). After removing duplicates (*n* = 338), 6,110 publications were evaluated based on their titles and abstracts. This evaluation led to the exclusion of 6,066 publications that did not meet the predefined eligibility criteria based on title and abstract. For the remaining 44 publications, the full text version was obtained and assessed using the same criteria.

**Fig. 1:**
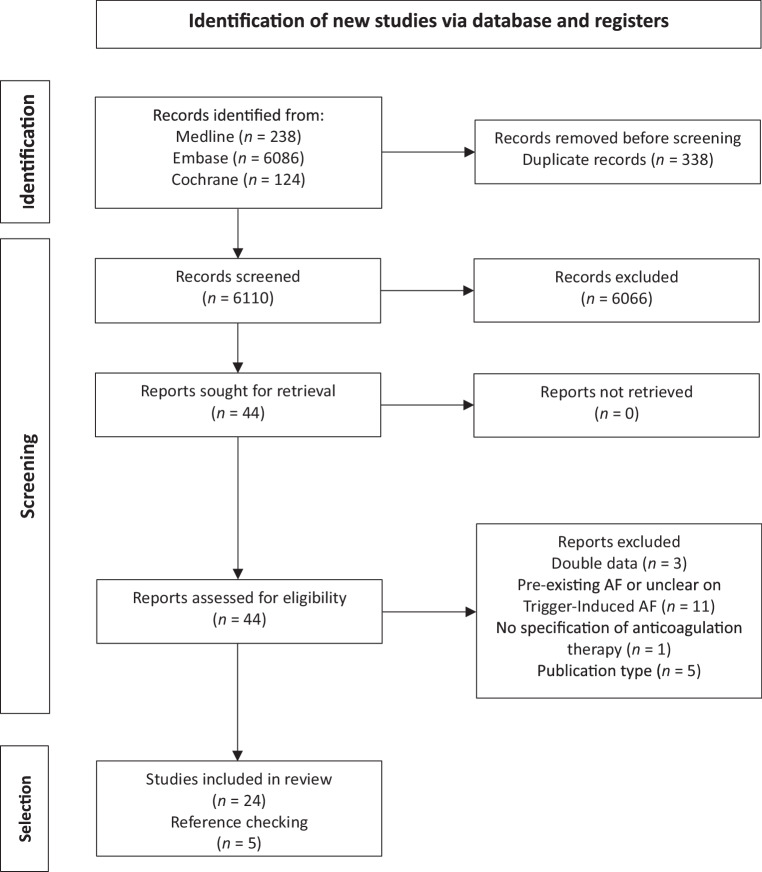
PRISMA flow diagram illustrating the identification, screening, eligibility, and inclusion process for the studies in the review

Of the remaining 44 publications, 24 were included in the review. An additional 5 publications were identified through relevant references from the full-text articles [18–22], resulting in a total of 29 publications included in the review (Fig. 1).

Two publications used the same dataset: one offered insight into anticoagulation prescriptions and CHA_2_DS_2_-VAScs, scores [23] while the other was included in the meta-analysis for its outcome data [24]. All included studies are summarized in table S2 and the risk of bias assessment is shown in table S3.

### Incidence of anticoagulation prescription

**Fig. 2:**
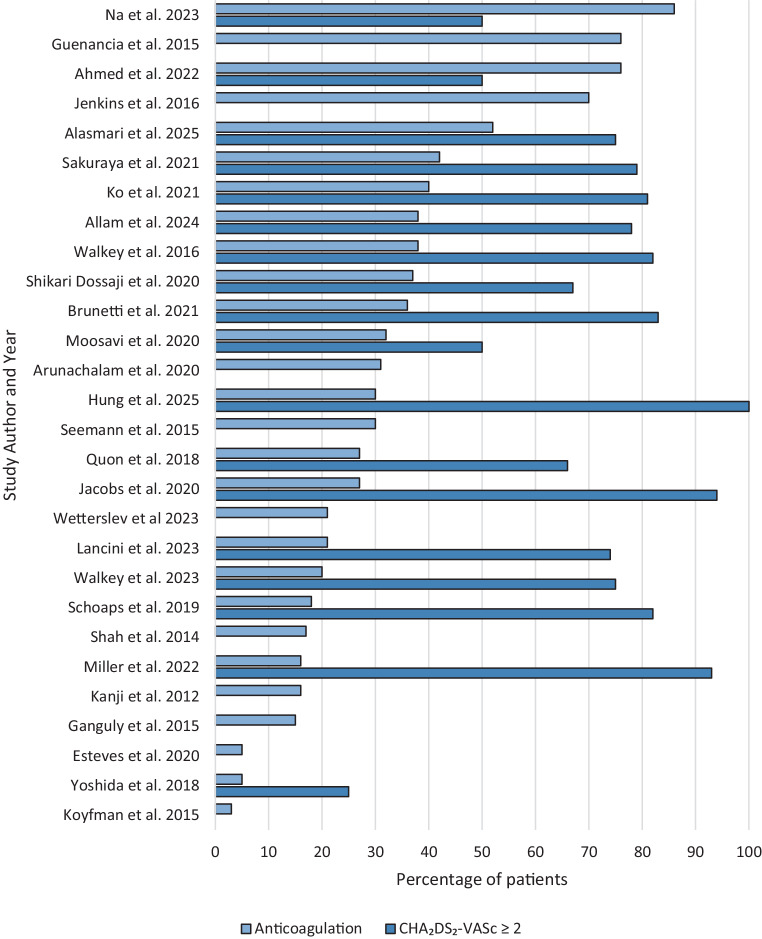
Anticoagulation rates and stroke risk based on CHA_2_DS_2_-VASc score. The studies are ranked according to the decreasing rate of anticoagulant medication. Upper bar: The proportion of patients in all studies included in the systematic review who were prescribed anticoagulation therapy for trigger-induced atrial fibrillation during acute hospitalisation. Lower bar: The proportion of patients with the reported or estimated CHA_2_DS_2_-VASc score of ≥ 2 in the corresponding publication. If unavailable the lower bar was not displayed

A total of 28 publications (excluding one publication with a duplicate dataset as previously mentioned) were identified as eligible for determining the incidence of therapeutic anticoagulation therapy in trigger-induced AF. Out of these, 13 reported on trigger-induced AF exclusively in sepsis patients. Anticoagulation prescription rates varied significantly, ranging from 3–86% in sepsis-only studies and 5–76% in the remaining studies (Fig. 2a).

### Estimation of CHA_2_DS_2_-VASc score and anticoagulation therapy

Ultimately, 18 studies provided sufficient predefined data on the CHA_2_DS_2_-VASc score, allowing us to estimate the proportion of patients with a CHA_2_DS_2_-VASc ≥ 2. Six studies reported exclusively on trigger-induced AF in sepsis patients. In all but one study [25] the estimated proportion of patients with CHA_2_DS_2_-VASc ≥ 2 was higher than the percentage of patients who were prescribed anticoagulation. This study by Ahmed et al. (2022) reported a median CHA_2_DS_2_-VASc score of 4 without providing other indices, limiting the ability to estimate a more exact CHA_2_DS_2_-VASc score, resulting in an estimation of 50% [25]. Overall, most studies included populations in which at least half of the patients had an estimated CHA_2_DS_2_-VASc ≥ 2. In these studies, the proportion of patients receiving anticoagulation therapy was lower than expected based on their CHA_2_DS_2_-VASc scores (Fig. 2b).

## Meta-analysis

A total of eight studies were identified that met the inclusion criteria. These studies stratified data by anticoagulation use, allowing outcome comparison in a 2 × 2 contingency table [11, 24, 26–31]. Seven of these studies reported data on thromboembolism and bleeding, while four studies reported mortality. Most of the included patients experienced sepsis or infection as the primary precipitating factor: 99 and 94% of the patients in short-term and long-term arms respectively (Table S2). None of the publications reported on the severity or sequelae of thromboembolism or bleeding. The overall incidence of thrombosis, bleeding, and mortality in every study is reported in table S2.

### Short-term outcomes

Four studies were included in the meta-analyses for short-term (all in-hospital outcome data) thrombosis and bleeding complications [11, 26, 30, 31]. The study by Walkey et al. also included patients with pre-existing AF and used propensity matching to compare anticoagulation vs. no anticoagulation in patients with similar risk factors [26]. Data from the propensity-matched cohort were used to calculate the number of events in the subset of trigger-induced AF patients. Details on data extraction and subsequent calculations are provided in table S4.

**Fig. 3:**
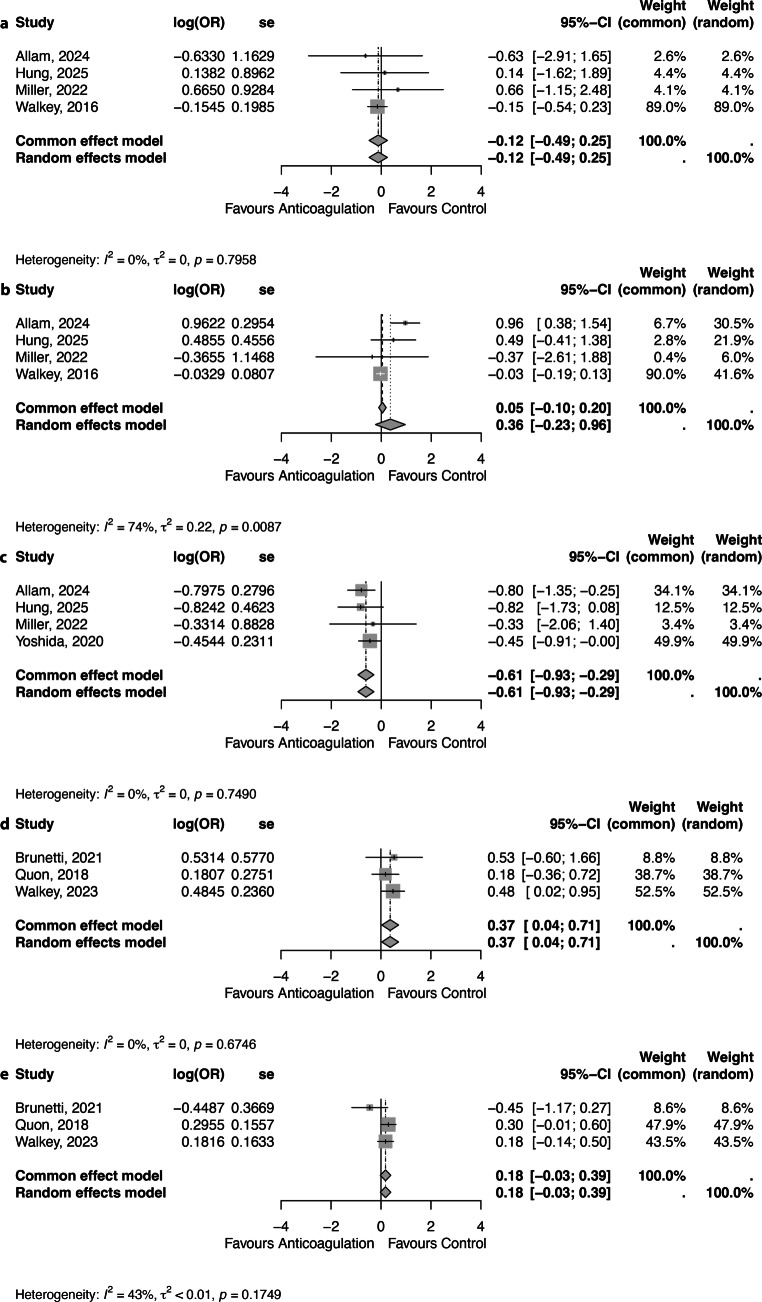
Forest plot showing the short-term **a** thromboembolism, **b** bleeding and **c** mortality and long-term **d** thromboembolism and **e** bleeding outcomes between anticoagulation therapy and control. OR – Odds Ratio, se – Standard Error, 95%CI – 95% Confidence Interval

The pooled association between anticoagulation and thrombotic complications was an OR of 0.89 (95% CI 0.61–1.28), with low between-study heterogeneity (I^2^ = 0%; Fig. 3a). The pooled association between anticoagulation and bleeding complications was an OR of 1.05 (95% CI 0.90–1.22), with considerable between-study heterogeneity (I^2^ = 74%; Fig. 3b).

Four studies were included in the meta-analysis for short-term (in-hospital) mortality [11, 24, 30, 31]. The pooled association between anticoagulation and mortality was an OR of 0.54 (95% CI 0.39–0.75), with low between-study heterogeneity (I^2^ = 0%; Fig. 3c).

### Long-term outcomes

The long-term outcomes for thromboembolism and bleeding were analysed based on three available studies: Brunetti et al. (2021), Quon et al. (2018), and Walkey et al. (2023) [27–29]. Quon et al. (2018) included a mixed cohort of acute pulmonary disease and sepsis patients, while Walkey et al. (2023) reported exclusively on sepsis patients [28, 29]. Follow-up in the studies varied from one [27, 29] to about three years [28].

The pooled association between anticoagulation and long-term thrombotic complications had an OR of 1.45 (95% CI 1.04–2.03), with low between-study heterogeneity (I^2^ = 0%; see Fig. 3d). When appraising the data from the per-protocol analysis from the study by Walkey et al. (2023) it yielded an OR of 1.80, (95% CI 1.28–2.56) (figure S1a).

The pooled association between anticoagulation and long-term bleeding complications had an OR of 1.20 (95% CI 0.97–1.48), with moderate between-study heterogeneity (I^2^ = 43%; Fig. 3e). When appraising the data from the per-protocol analysis from the study by Walkey et al. (2023), it yielded an OR of 1.09 (95% CI 0.87–1.38) (figure S1b). There was no available outcome data on long-term mortality.

## Discussion

This review and meta-analysis demonstrated substantial variation in anticoagulation prescription in critically ill patients with trigger-induced AF and low adherence to CHA_2_DS_2_-VASc. The meta-analysis demonstrated no association between anticoagulation prescription and thromboembolism (or stroke) reduction in patients with trigger-induced AF. Notably, the use of anticoagulation was associated with improved short-term survival but an increased incidence of long-term stroke, while the incidence of bleeding was not influenced.

The decision to initiate anticoagulation is preceded by a careful weighing of the benefits (prevention of thromboembolism) and risks (increased bleeding risk). Ideally, the decision is informed by the baseline risks and the relative effect of anticoagulation on these outcomes. However, in trigger-induced AF, the CHA_2_DS_2_-VASc score has been shown to perform poorly in predicting stroke risk [9]. Additionally, the available data from the included studies did not allow us to assess any association between the CHA_2_DS_2_-VASc score and stroke risk.

We found that the association between anticoagulation and the risk of in-hospital thromboembolic events was small in both relative and absolute terms. We identified no statistically significant association between anticoagulation and in-hospital thromboembolism. The outer limit of the 95% confidence interval (an OR of 0.61) would constitute an absolute risk difference of merely 0.8% in favour of anticoagulation at an average baseline thromboembolic risk of approximately 2% during hospitalisation [26]. This should be weighed against the risk of bleeding. Although we also found no association between anticoagulation and in-hospital bleeding, the uncertainty in terms of absolute risks is higher because clinically significant bleeding is much more common than thromboembolism. The outer limit of the confidence interval (OR 1.22) would constitute an absolute risk increase of 2.8% at an average baseline bleeding risk of approximately 12.6%. It is possible that some deaths may have resulted from fatal strokes or bleeding, which might have been missed due to the observational nature of these studies.

These findings persist despite the implementation of methods such as propensity score matching and sensitivity analyses to address potential biases in several studies. Likely, the observed associations do not represent the unbiased causal effects of anticoagulation [9, 26, 29]. Yet even in the face of potential biases, the results indicate, on the whole, that the conventional risk-benefit analysis is not applicable in trigger-induced AF during acute hospitalisation. The high absolute risk of bleeding and the high relative risk of stroke under anticoagulation (i.e., the apparent lack of protective effect) are profoundly different from patients with ambulant AF. Appropriate and validated stratification of stroke and bleeding risk for this population is an important knowledge gap.

The retrospective nature of the included studies introduces several types of bias, most notably confounding by indication. Patients at a perceived increased risk of thromboembolism or stroke or with a high AF burden are more likely to be prescribed anticoagulation therapy. This is one of the likely explanations for the association between anticoagulation use and long-term thrombotic complications. Also, the adequacy of anticoagulation therapy remains uncertain due to the lack of data regarding patient adherence, the presence of treatment interruptions, maintenance of therapeutic INR levels, or the appropriateness of DOAC dosing.

Immortal time bias is potentially introduced as clinicians may choose to delay anticoagulation therapy and thereby increasing mortality in non-treated patients as a result of early deaths [32–34]. Also, if a patient survives, sinus rhythm restoration may impact the decision regarding long-term anticoagulation This form of AF, which appears in the context of critical illness, is potentially transient [19, 35], and overall AF burden is reported to be relatively low [36–38], with about two-thirds of patients experience AF for less than 24 h [1, 19, 35].

Long-term recurrence data indicate that about one-third to half of patients experience AF within one year of hospitalisation [36–38]. Its prevalence may be underestimated, and intensive monitoring increases the likelihood of detecting recurrent AF [39]. The decision to initiate, continue, or terminate anticoagulation should be guided by the actual recurrence of AF and its associated AF burden, not merely by the occurrence of a single AF event. Andreotti and De Caterina have proposed such an approach for post-CABG AF [40, 41]. Here, advances in the clinical quality of care can be made even without randomized trials. Extensive monitoring can provide better insight into which patients have recurrent AF, and this should be easier with recent technological advances.

### Limitations and future perspectives

First, due to the paucity of data with a high potential for bias, only three or four eligible studies were identified for each outcome (thromboembolism, bleeding, mortality) and time frame (short-term and long-term). Second, heterogeneity was present, as the patient population and follow-up varied considerably between publications. Although most patients were admitted with sepsis or infection (approximately 95%), a proportion of those classified as critically ill had no documented underlying disease. Third, no publications included in the meta-analysis reported on the severity or sequelae of thrombotic and bleeding events, precluding a more detailed assessment of the overall risk-benefit profile of anticoagulation. Fourth, and of importance, there is no clear standardized definition of trigger-induced AF or secondary AF. Recently, the term trigger-induced AF has been adopted by the 2024 European guidelines, and ‘acute AF’ was used in a scientific statement of the AHA. It was defined as “AF detected in an acute care setting or during an acute illness” [2, 4]. This term was explicitly introduced to avoid the need to determine if the atrial fibrillation is truly (temporarily) triggered by the acute illness or has simply been undiagnosed until that moment. This dilemma is applicable to most patients in the existing literature and consequently to our current analysis.

No randomized controlled trials are underway that address anticoagulation in patients with trigger-induced AF, both for the short term (during hospitalisation) and the long term (after discharge). Due to the low incidence of the primary endpoints, such trials will require the enrolment of thousands of patients and may not be forthcoming in the foreseeable future. More importantly, due to the heterogeneity of acutely hospitalised patients, no single best strategy for all trigger-induced AF cases will likely ever be identified. To make meaningful progress, we should first prioritize prediction and risk stratification.

Future research should focus on the predictive value of a prolonged in-hospital or post-discharge monitoring period for long-term AF recurrence. Ultimately, a comprehensive approach to decision-making should also incorporate thromboembolic risk stratification. For now, it remains unclear if the CHA_2_DS_2_-VASc score is a reliable predictor of thromboembolic complications in this patient category. Efforts should be made to either validate or improve the performance of available risk scores. This may help to avoid exposing patients with low thrombotic risk and those without recurrent AF to anticoagulants.

## Conclusion

In critically ill patients with trigger-induced atrial fibrillation, prescription of anticoagulation is highly variable. No association between anticoagulation prescription and altered short-term thromboembolism or bleeding incidence was demonstrated. While the use of anticoagulation was associated with improved short-term survival, it was paradoxically associated with a higher incidence of long-term stroke, while bleeding rates were unaffected. This study neither confirms nor excludes the potential benefits or harms of anticoagulation. Its use should therefore be decided on a case-by-case basis.

## Supplementary Information

Table S1: Search Strategy

Table S2: Overview of included publications

Table S3: New Castle Ottowa Scale (NOS) Risk of Bias Assessment

Table S4: Calculations and contingency tables

Figure S1a: Forest plot showing long-term thromboembolism outcomes between anticoagulation therapy and control. Results from Walkey et al. (2023) are per protocol outcomes. OR Odds Ratio, se Standard Error, 95% CI 95% Confidence Interval

Figure S1b: Forest plot showing long-term bleeding outcomes between anticoagulation therapy and control. Results from Walkey et al. (2023) are per protocol outcomes. OR Odds Ratio, se Standard Error, 95% CI 95% Confidence Interval
