# Correction to: Anticoagulation and thromboembolic risk in critically ill patients with trigger-induced atrial fibrillation—A systematic review and meta-analysis

**DOI:** 10.1007/s12471-025-01991-y

**Published:** 2025-09-17

**Authors:** Jasper Koolwijk, Mileen van de Kar, Brittney A van der Woude, Marcel van ’t Veer, Harm Jan de Grooth, Harry J. G. M. Crijns, Lukas R. C. Dekker, R. Arthur Bouwman, Olaf L. Cremer, Ashley J. R. de Bie, Luuk C. Otterspoor

**Affiliations:** 1 Department of Anesthesiology, Intensive Care and Pain Medicine, Catharina Hospital Eindhoven, Eindhoven, The Netherlands; 2 Department of Cardiology and Cardiothoracic Surgery, Catharina Hospital Eindhoven, Eindhoven, The Netherlands; 3 Intensive Care Center, University Medical Center Utrecht, Utrecht, The Netherlands; 4 Department of Cardiology, Maastricht University Medical Center, Cardiovascular Research Institute Maastricht, Maastricht, The Netherlands; 5 Department of Electrical Engineering, Eindhoven University of Technology, Eindhoven, The Netherlands; 6 Department of Intensive Care, Catharina Hospital Eindhoven, Eindhoven, The Netherlands

**Correction to:**

**Neth Heart J 2025**

10.1007/s12471-025-01978-9

The version of Fig. 3 published in the original article was incorrect and has now been replaced by an amended version. The caption and any mentions of Fig. 3 within the text have also been corrected to reflect this change. The original article has been corrected.

**Fig. 3 - Incorrect:**
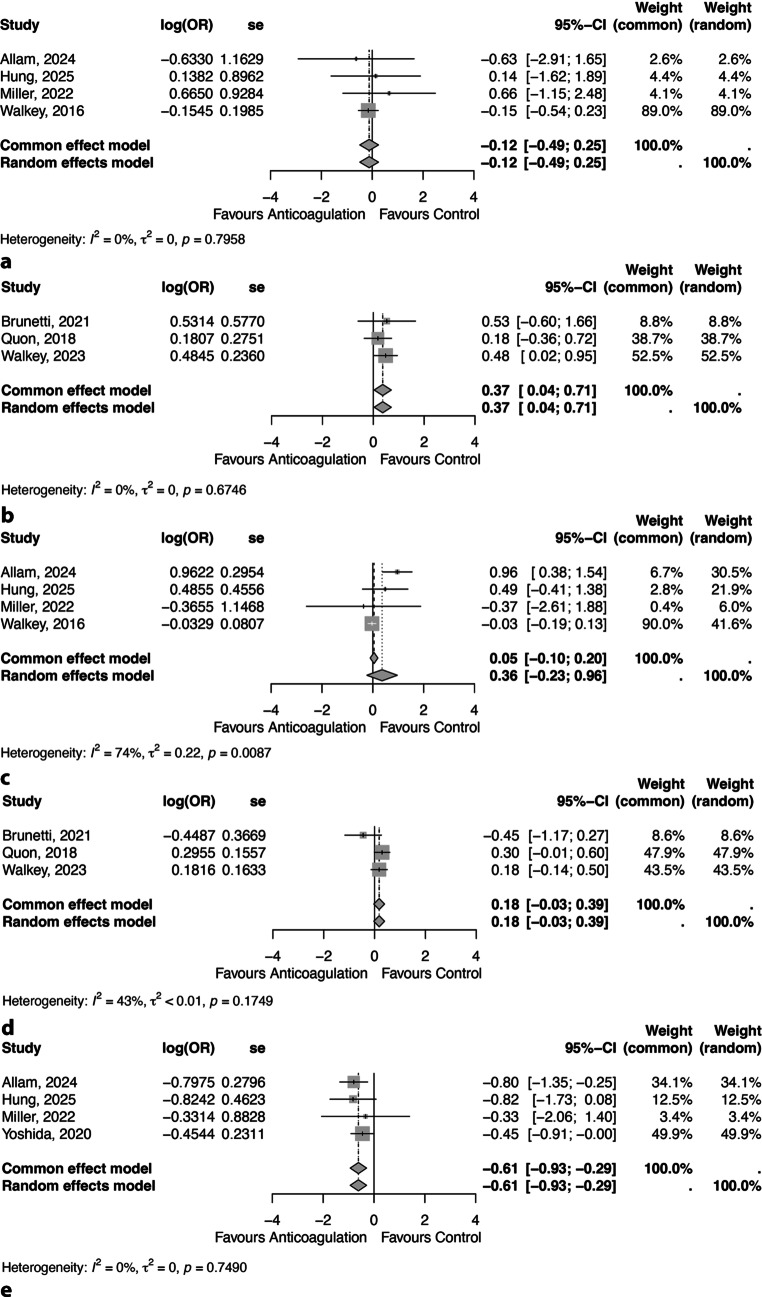
Forest plot showing the short-term **a** thromboembolism, **b** thromboembolism, **c** bleeding, **d** bleeding outcomes between anticoagulation therapy and control and **e** mortality and long-term. *OR* Odds Ratio, *se* Standard Error, *95%CI* - 95% Confidence Interval

**Fig. 3 - Correct:**
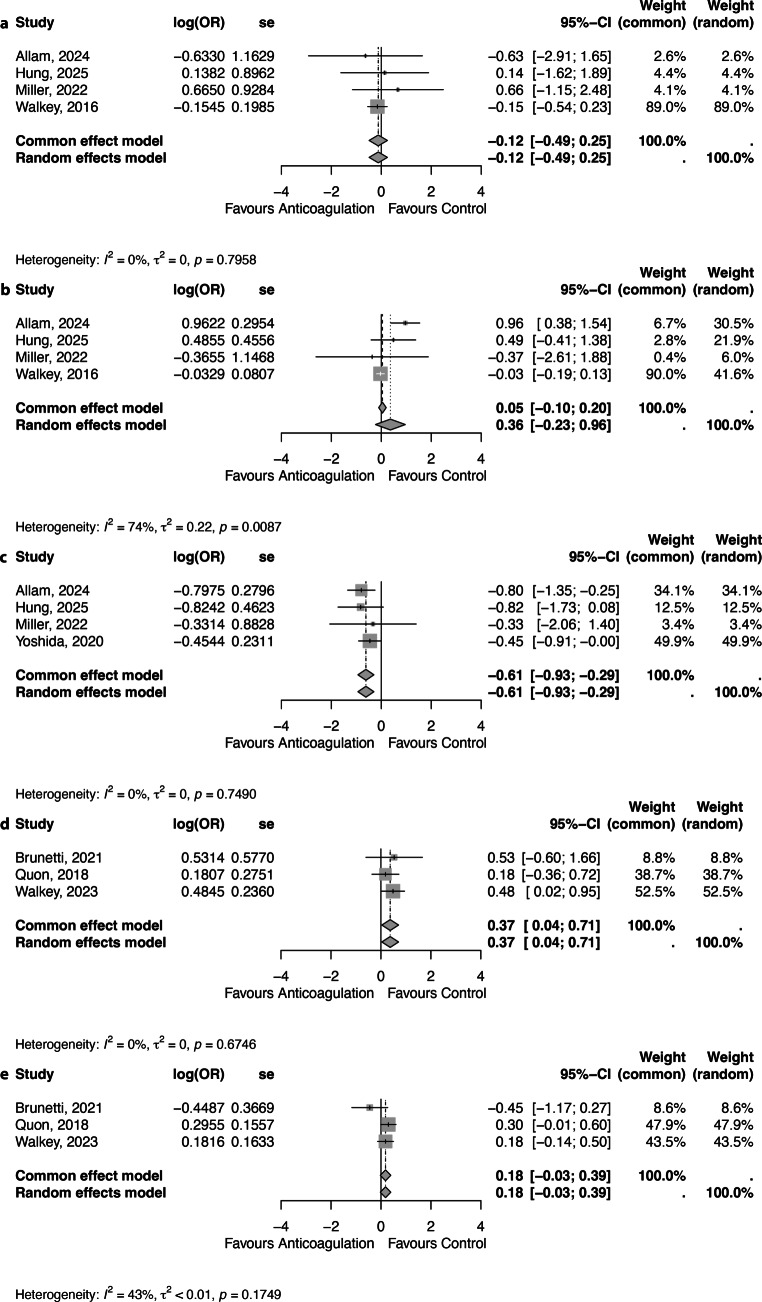
Forest plot showing the short-term **a** thromboembolism, **b** bleeding and **c** mortality and long-term **d** thromboembolism and **e** bleeding outcomes between anticoagulation therapy and control. OR – Odds Ratio, se – Standard Error, 95%CI – 95% Confidence Interval

