# Supplementary material for: Anticoagulation and thromboembolic risk in critically ill patients with trigger-induced atrial fibrillation—A systematic review and meta-analysis
Source: Neth Heart J. 2025 Aug 28;33(10):290–8. doi: 10.1007/s12471-025-01978-9 (PMC12454756; doi:10.1007/s12471-025-01978-9)
Supplement: Supplementary file 1 — Table S1: Search Strategy [file 12471_2025_1978_MOESM1_ESM.docx]

| **Search Strategy**  Last performed on 31/03/2025 |
| --- |
| **Medline:**  ((("Sepsis"[Mesh]) OR "sepsis" [tiab] OR "septic*" [tiab]) OR ((("Critical Care"[Mesh]) OR "Intensive Care Units"[Mesh]) OR ("critical care"[tiab] OR "Intensive care"[tiab] OR "critical* ill*"[tiab] OR "high depend*"[tiab]))) AND (("Atrial Fibrillation"[Mesh]) OR "atrial fibrillation"[tiab] OR "atrium fibrillation"[tiab] OR "new onset atrial fibrillation"[tiab] OR "NOAF"[tiab]) AND (("Anticoagulants"[Mesh] OR "Heparin"[Mesh] OR "Heparin, Low-Molecular-Weight"[Mesh] OR "Rivaroxaban"[Mesh] OR "Dabigatran"[Mesh]) OR "anticoagulant*" [tiab] OR "anticoagulation" [tiab] OR "oral anticoagulation" [tiab] OR "anticoagulant therapy" [tiab] OR "oral anticoagulant therapy" [tiab] OR "anticoagulant agents" [tiab] OR "anticoagulant drugs" [tiab] OR "OAC" [tiab]) AND (("Mortality"[Mesh]) OR "death" [tiab] OR "mortality" [tiab] OR ("Stroke"[Mesh]) OR "stroke" [tiab] OR "cerebrovascular accident" [tiab] OR "CVA" [tiab] OR ("Hemorrhage"[Mesh]) OR "bleeding" [tiab] OR "hemorrhage" [tiab] OR "major bleeding" [tiab] OR "anticoagulant-related bleeding" [tiab]) |
| **Embase:**  ((('sepsis'/exp) OR 'sepsis':ti,ab,kw OR 'septic*':ti,ab,kw) OR ((('intensive care'/exp) OR 'intensive care unit'/exp) OR ('critical care':ti,ab,kw OR 'intensive care':ti,ab,kw OR 'critical* ill*':ti,ab,kw OR 'high depend*':ti,ab,kw))) AND (('atrial fibrillation'/exp) OR 'atrial fibrillation':ti,ab,kw OR 'atrium fibrillation':ti,ab,kw OR 'new onset atrial fibrillation':ti,ab,kw OR 'noaf':ti,ab,kw) AND (('anticoagulant agent'/exp OR 'heparin'/exp OR 'low molecular weight heparin'/exp OR 'rivaroxaban'/exp OR 'dabigatran'/exp) OR 'anticoagulant*':ti,ab,kw OR 'anticoagulation':ti,ab,kw OR 'oral anticoagulation':ti,ab,kw OR 'anticoagulant therapy':ti,ab,kw OR 'oral anticoagulant therapy':ti,ab,kw OR 'anticoagulant agents':ti,ab,kw OR 'anticoagulant drugs':ti,ab,kw OR 'oac':ti,ab,kw) AND (('mortality'/exp) OR 'death':ti,ab,kw OR 'mortality':ti,ab,kw OR ('cerebrovascular accident'/exp) OR 'stroke':ti,ab,kw OR 'cerebrovascular accident':ti,ab,kw OR 'cva':ti,ab,kw OR ('bleeding'/exp) OR 'bleeding':ti,ab,kw OR 'hemorrhage':ti,ab,kw OR 'major bleeding':ti,ab,kw OR 'anticoagulant-related bleeding':ti,ab,kw) |
| **Cochrane:**  ID Search  #1 MeSH descriptor: [Sepsis] explode all trees  #2 (sepsis):ti,ab,kw  #3 (septicemia):ti,ab,kw  #4 (septic*):ti,ab,kw  #5 MeSH descriptor: [Critical Care] explode all trees  #6 (critical care):ti,ab,kw  #7 (intensive care):ti,ab,kw  #8 (high depen*):ti,ab,kw  #9 MeSH descriptor: [Critical Care] explode all trees  #10 (critical* ill):ti,ab,kw  #11 #1 OR #2 OR #3 OR #4 OR #5 OR #6 OR #7 OR #8 OR #9 OR #10  #12 MeSH descriptor: [Atrial Fibrillation] explode all trees  #13 (atrial fibrillation):ti,ab,kw  #14 (atrium fibrillation):ti,ab,kw  #15 (NOAF):ti,ab,kw  #16 #13 OR #14 OR #15  #17 MeSH descriptor: [Anticoagulants] explode all trees  #18 MeSH descriptor: [Heparin] explode all trees  #19 MeSH descriptor: [Heparin, Low-Molecular-Weight] explode all trees  #20 (anticoagulants):ti,ab,kw  #21 (heparin):ti,ab,kw  #22 (low molecular weight heparin):ti,ab,kw  #23 (LMWH):ti,ab,kw  #24 (rivaroxaban):ti,ab,kw  #25 (apixaban):ti,ab,kw  #26 (edoxaban):ti,ab,kw  #27 (dabigatran):ti,ab,kw  #28 ("anticoagulant therapies"):ti,ab,kw  #29 ("anticoagulant therapy"):ti,ab,kw  #30 (oral anticoagulation):ti,ab,kw  #31 (OAC):ti,ab,kw  #32 #17 OR #18 OR #19 OR #20 OR #21 OR #22 OR #23 OR #24 OR #25 OR #26 OR #27 OR #28 OR #29 OR #30 OR #31  #33 MeSH descriptor: [Mortality] explode all trees  #34 (mortality):ti,ab,kw  #35 MeSH descriptor: [Stroke] explode all trees  #36 (stroke):ti,ab,kw  #37 (cerebrovascular accident):ti,ab,kw  #38 (CVA):ti,ab,kw  #39 MeSH descriptor: [Hemorrhage] explode all trees  #40 (bleeding*):ti,ab,kw  #41 (hemorrhage*):ti,ab,kw  #42 (major bleeding):ti,ab,kw  #43 (anticoagulant-related bleeding):ti,ab,kw  #44 #33 OR #34 OR #35 OR #36 OR #37 OR #38 OR #39 OR #40 OR #41 OR #42 OR #43  #45 #11 AND #16 AND #32 AND #44 |
