# Supplementary material for: Anticoagulation and thromboembolic risk in critically ill patients with trigger-induced atrial fibrillation—A systematic review and meta-analysis
Source: Neth Heart J. 2025 Aug 28;33(10):290–8. doi: 10.1007/s12471-025-01978-9 (PMC12454756; doi:10.1007/s12471-025-01978-9)
Supplement: Supplementary file 4 — Table S4: Calculations and contingency tables [file 12471_2025_1978_MOESM4_ESM.docx]

| **Article** | **Data source** |
| --- | --- |
| Allam 2024 | Table 4 |
| Hung 2025 | Table 3 |
| Miller 2022 | Figure 1, Table 6 |
| Walkey 2016 | Source:   - Table 1 – total number of patients with new onset atrial fibrillation calculated as total number of patients -/- patients with pre-exisiting atrial fibrillation - Results section “Outcomes Stratified by Newly Diagnosed and Preexisting AF” –   Total number of events in the new-onset group were combined with the reported RR and calculated backward. Rounded to nearest integer.  **Calculations**  **Stroke: RR = 0.85 (95% CI 0.57-1.17)**  Treatment arm: 2789 = a+b No treatment arm: 2796 = = c+d Disease positive total: 104 = a+c Disease negative total 5481 = =b+d Total: 5585  RR = (a / ( a + b ) ) / (c / ( c + d ) ) RR = (a / 2789) / ((104 - a) / 2796) = 0.85 0.85 * 2789 * (104 - a) = a * 2796 2360.65 * (104 - a) = a * 2796 a ≈ 48 c = 104 - 48 = 56 b = 2789 - 48 = 2741 d = 5481 - 2741 = 2740  **Bleeding: RR = 0.97 (95% CI 0.83-1.14)**  Treatment positive: 2789 a + b Treatment negative: 2796 c + d Disease positive: 703 a + c Disease negative: 4882 b + d Total: 5585  RR = (a / ( a + b ) ) / (c / ( c + d ) ) RR = (a / 2789) / ((703 - a) / 2796) = 0.97 0.97 * 2789 * (703 - a) = a * 2796 2705.33 * (703 - a) = a * 2796 a ≈ 346 c = 703 - 346 = 357 b = 2789 - 346 = 2443 d = 4882 - 2443 = 2439 |
| Yoshida 2020 | Table 4 – direct data |
| Brunetti 2021 | Table 3 – direct data |
| Quon 2018 | Table 2 and Table 3 – direct data |
| Walkey 2023 | Total data on number of events and censored subjects were combined with the reported percentages and calculated backward. Rounded to nearest integer.  Calculations  OAC Prescription: 807 patients  No OAC Prescription: 3185 patients  **Overall Approach (Intention-to-Treat)**  Given percentages:  Stroke/TIA with OAC: 3.2%  Stroke/TIA without OAC: 2.0%  Bleeding with OAC: 6.4%  Bleeding without OAC: 5.4%  Stroke/TIA with OAC:  Patients = 807 * 3.2 / 100 = 25.824 ≈ 26 patients  Stroke/TIA without OAC:  Patients = 3185 * 2.0 / 100 = 63.7 ≈ 64 patients  Bleeding with OAC:  Patients = 807 * 6.4 / 100 = 51.648 ≈ 52 patients  Bleeding without OAC:  Patients = 3185 * 5.4 / 100 = 172.995 ≈ 173 patients  **Per-Protocol Approach**  Total numbers:  OAC Prescription: 422 patients (385 censored)  No OAC Prescription: 2647 patients (538 censored)  Given percentages:  Stroke/TIA with OAC: 5.69%  Stroke/TIA without OAC: 2.32%  Bleeding with OAC: 6.51%  Bleeding without OAC: 7.10%  Calculations:  Stroke/TIA with OAC:  Patients = 422 * 5.69 / 100 = 24.00 ≈ 24 patients  Stroke/TIA without OAC:  Patients = 2647 * 2.32 / 100 = 61.45 ≈ 61 patients  Bleeding with OAC:  Patients = 422 * 6.51 / 100 = 27.48 ≈ 27 patients  Bleeding without OAC:  Patients = 2647 * 7.10 / 100 = 187.94 ≈ 188 patients |

**Strokest**

Study 1: Allam (2024)

|  | Treatment | Control | Total |
| --- | --- | --- | --- |
| Positive | 1 | 3 | 4 |
| Negative | 86 | 137 | 223 |
| Total | 87 | 140 | 227 |

Study 2: Hung (2025)

|  | Treatment | Control | Total |
| --- | --- | --- | --- |
| Positive | 2 | 4 | 6 |
| Negative | 27 | 62 | 89 |
| Total | 29 | 66 | 95 |

Study 3: Miller (2022)

|  | Treatment | Control | Total |
| --- | --- | --- | --- |
| Positive | 2 | 6 | 8 |
| Negative | 6 | 35 | 41 |
| Total | 8 | 41 | 49 |

Study 4: Walkey (2016)

|  | Treatment | Control | Total |
| --- | --- | --- | --- |
| Positive | 48 | 56 | 104 |
| Negative | 2741 | 2740 | 5481 |
| Total | 2789 | 2796 | 5585 |

**Bleedst**

Study 1: Allam (2024)

|  | Treatment | Control | Total |
| --- | --- | --- | --- |
| Positive | 38 | 32 | 70 |
| Negative | 49 | 108 | 157 |
| Total | 87 | 140 | 227 |

Study 2: Hung (2025)

|  | Treatment | Control | Total |
| --- | --- | --- | --- |
| Positive | 13 | 22 | 35 |
| Negative | 16 | 44 | 60 |
| Total | 29 | 66 | 85 |

Study 3: Miller (2022)

|  | Treatment | Control | Total |
| --- | --- | --- | --- |
| Positive | 1 | 7 | 8 |
| Negative | 7 | 34 | 41 |
| Total | 8 | 41 | 49 |

Study 4: Walkey (2016)

|  | Treatment | Control | Total |
| --- | --- | --- | --- |
| Positive | 346 | 357 | 703 |
| Negative | 2443 | 2439 | 4882 |
| Total | 2789 | 2796 | 5585 |

**mortst**

Study 1: Allam (2024)

|  | Treatment | Control | Total |
| --- | --- | --- | --- |
| Positive | 41 | 93 | 134 |
| Negative | 46 | 47 | 93 |
| Total | 87 | 140 | 227 |

Study 2: Hung (2025)

|  | Treatment | Control | Total |
| --- | --- | --- | --- |
| Positive | 10 | 36 | 46 |
| Negative | 19 | 30 | 49 |
| Total | 29 | 66 | 95 |

Study 3: Miller (2022)

|  | Treatment | Control | Total |
| --- | --- | --- | --- |
| Positive | 2 | 13 | 15 |
| Negative | 6 | 28 | 34 |
| Total | 8 | 41 | 49 |

Study 4: Yoshida (2020)

|  | Treatment | Control | Total |
| --- | --- | --- | --- |
| Positive | 37 | 75 | 112 |
| Negative | 136 | 175 | 311 |
| Total | 173 | 250 | 423 |

**strokelt**

Study 1: Brunetti (2021)

|  | Treatment | Control | Total |
| --- | --- | --- | --- |
| Positive | 7 | 6 | 13 |
| Negative | 72 | 105 | 177 |
| Total | 79 | 111 | 190 |

Study 2: Quon (2018)

|  | Treatment | Control | Total |
| --- | --- | --- | --- |
| Positive | 22 | 37 | 59 |
| Negative | 469 | 945 | 1414 |
| Total | 491 | 982 | 1473 |

Study 3: Walkey (2023)

|  | Treatment | Control | Total |
| --- | --- | --- | --- |
| Positive | 26 | 63 | 89 |
| Negative | 781 | 3122 | 3903 |
| Total | 807 | 3185 | 3992 |

**bleedlt**

Study 1: Brunetti (2021)

|  | Treatment | Control | Total |
| --- | --- | --- | --- |
| Positive | 14 | 28 | 42 |
| Negative | 65 | 83 | 148 |
| Total | 79 | 111 | 190 |

Study 2: Quon (2018)

|  | Treatment | Control | Total |
| --- | --- | --- | --- |
| Positive | 79 | 125 | 204 |
| Negative | 412 | 876 | 1288 |
| Total | 491 | 1001 | 1492 |

Study 3: Walkey (2023)

|  | Treatment | Control | Total |
| --- | --- | --- | --- |
| Positive | 52 | 173 | 225 |
| Negative | 755 | 3012 | 3767 |
| Total | 807 | 3185 | 3992 |

**strokeltpp**

Study 1: Brunetti (2021)

|  | Treatment | Control | Total |
| --- | --- | --- | --- |
| Positive | 7 | 6 | 13 |
| Negative | 72 | 105 | 177 |
| Total | 79 | 111 | 190 |

Study 2: Quon (2018)

|  | Treatment | Control | Total |
| --- | --- | --- | --- |
| Positive | 22 | 37 | 59 |
| Negative | 469 | 945 | 1414 |
| Total | 491 | 982 | 1473 |

Study 3: Walkey (2023)

|  | Treatment | Control | Total |
| --- | --- | --- | --- |
| Positive | 24 | 61 | 85 |
| Negative | 398 | 2586 | 2984 |
| Total | 422 | 2647 | 3069 |

**bleedltpp**

Study 1: Brunetti (2021)

|  | Treatment | Control | Total |
| --- | --- | --- | --- |
| Positive | 14 | 28 | 42 |
| Negative | 65 | 83 | 148 |
| Total | 79 | 111 | 190 |

Study 2: Quon (2018)

|  | Treatment | Control | Total |
| --- | --- | --- | --- |
| Positive | 79 | 125 | 204 |
| Negative | 412 | 876 | 1288 |
| Total | 491 | 1001 | 1492 |

Study 3: Walkey (2023)

|  | Treatment | Control | Total |
| --- | --- | --- | --- |
| Positive | 27 | 188 | 215 |
| Negative | 395 | 2459 | 2854 |
| Total | 422 | 2647 | 3069 |

**strokelt2**

Study 1: Quon (2018)

|  | Treatment | Control | Total |
| --- | --- | --- | --- |
| Positive | 22 | 37 | 59 |
| Negative | 469 | 945 | 1414 |
| Total | 491 | 982 | 1473 |

Study 2: Walkey (2023)

|  | Treatment | Control | Total |
| --- | --- | --- | --- |
| Positive | 26 | 63 | 89 |
| Negative | 781 | 3122 | 3903 |
| Total | 807 | 3185 | 3992 |

**bleedlt2**

Study 1: Quon (2018)

|  | Treatment | Control | Total |
| --- | --- | --- | --- |
| Positive | 79 | 125 | 204 |
| Negative | 412 | 876 | 1288 |
| Total | 491 | 1001 | 1492 |

Study 2: Walkey (2023)

|  | Treatment | Control | Total |
| --- | --- | --- | --- |
| Positive | 52 | 173 | 225 |
| Negative | 755 | 3012 | 3767 |
| Total | 807 | 3185 | 3992 |
