# Supplementary figures and images for: Anticoagulation and thromboembolic risk in critically ill patients with trigger-induced atrial fibrillation—A systematic review and meta-analysis
Source: Neth Heart J. 2025 Aug 28;33(10):290–8. doi: 10.1007/s12471-025-01978-9 (PMC12454756; doi:10.1007/s12471-025-01978-9)

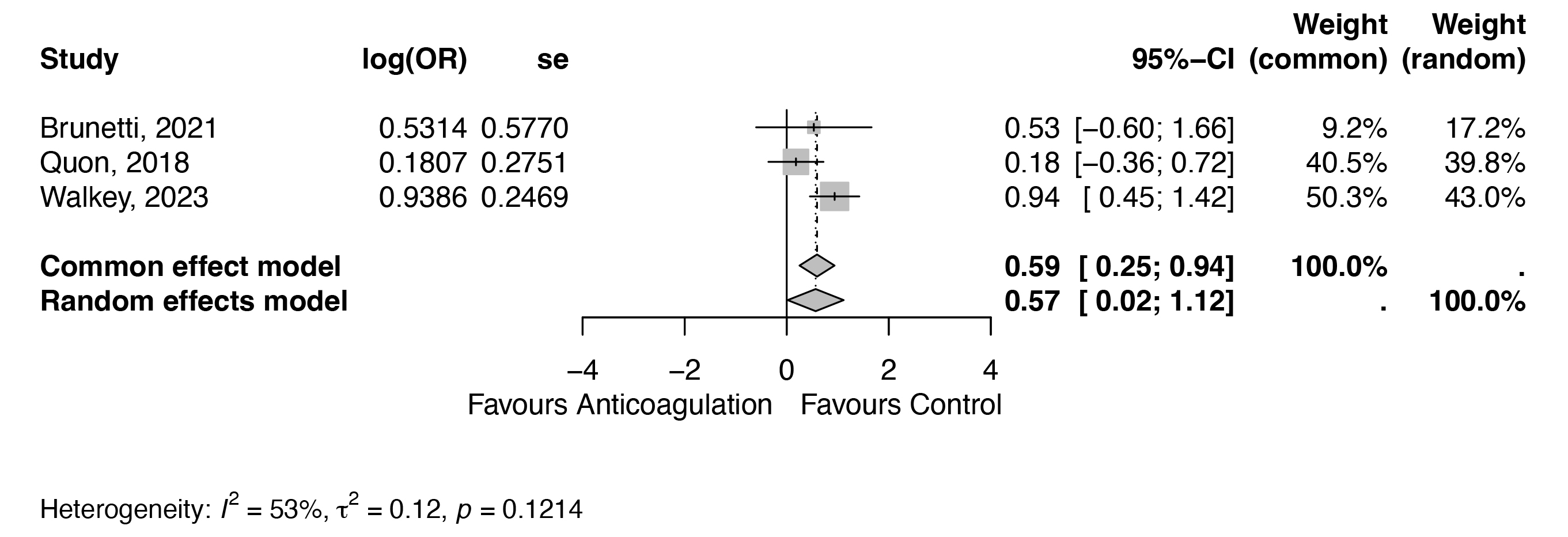

Supplement: Supplementary file 5 — Figure S1a: Forest plot showing long-term thromboembolism outcomes between anticoagulation therapy and control. Results from Walkey et al. (2023) are per protocol outcomes. OR Odds Ratio, se Standard Error, 95% CI 95% Confidence Interval [file 12471_2025_1978_MOESM5_ESM.jpg]

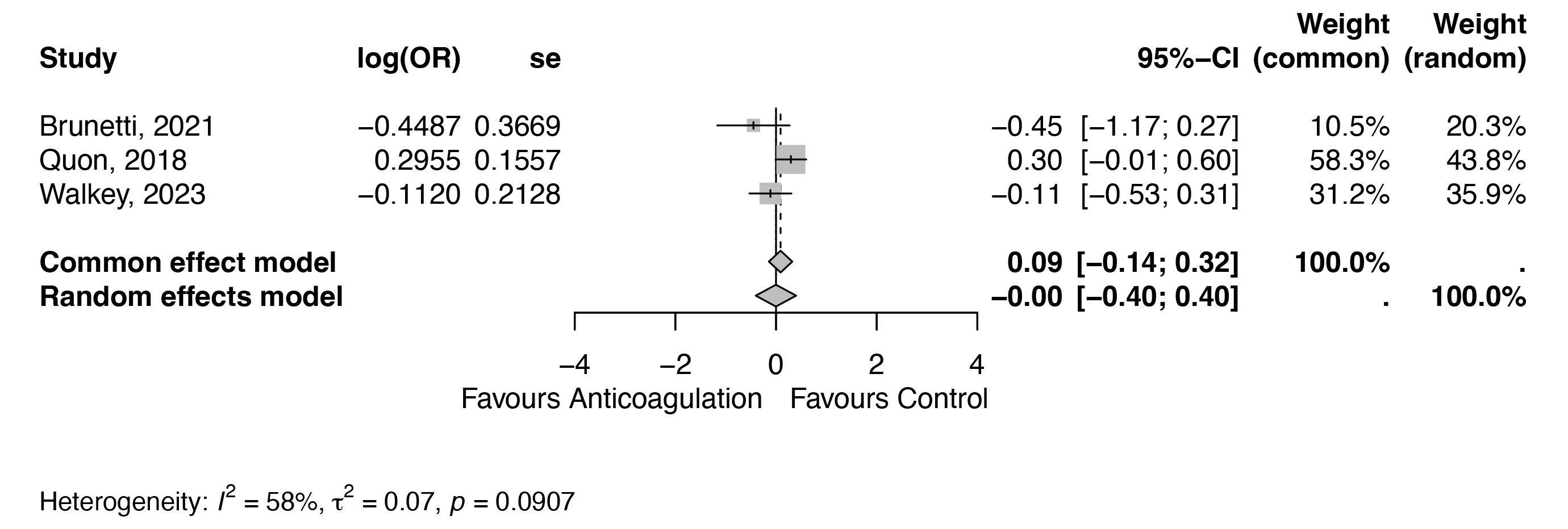

Supplement: Supplementary file 6 — Figure S1b: Forest plot showing long-term bleeding outcomes between anticoagulation therapy and control. Results from Walkey et al. (2023) are per protocol outcomes. OR Odds Ratio, se Standard Error, 95% CI 95% Confidence Interval [file 12471_2025_1978_MOESM6_ESM.jpg]
